# Supplementary material for: The interactome of intact mitochondria by cross-linking mass spectrometry provides evidence for coexisting respiratory supercomplexes
Source: Mol Cell Proteomics. 2017 Dec 8;17(2):216–32. doi: 10.1074/mcp.RA117.000470 (PMC5795388; doi:10.1074/mcp.RA117.000470)
Supplement: Supplemental Data [file supp_RA117.000470_133922_0_supp_23978_fzffwf.zip › spectra_annotation/mito_spectra_annotation/479-2-2-1-9-1.pdf]

VLAKLAEK
LVFTPKDGSSAK

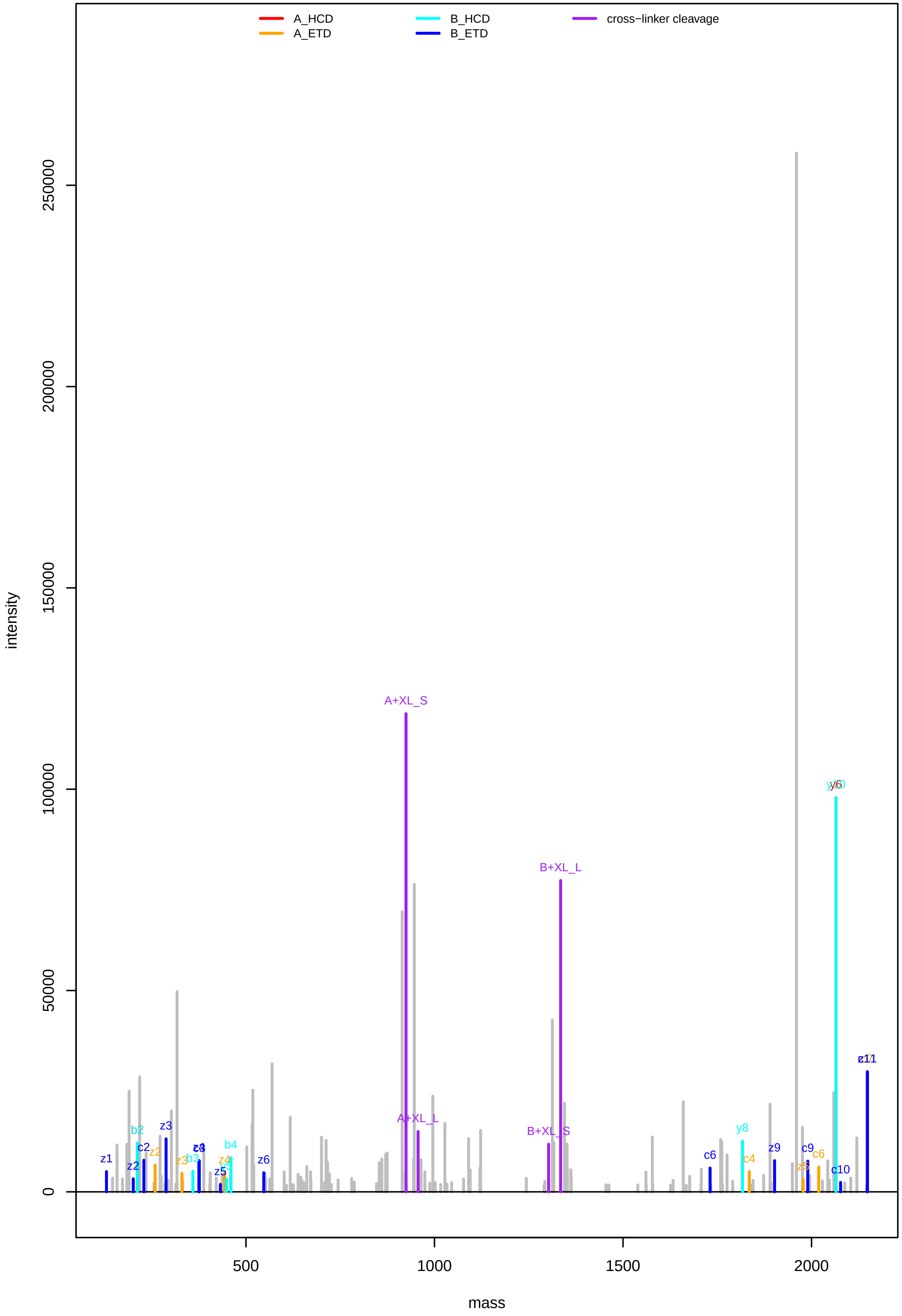

VLAKLAEK+XL_S

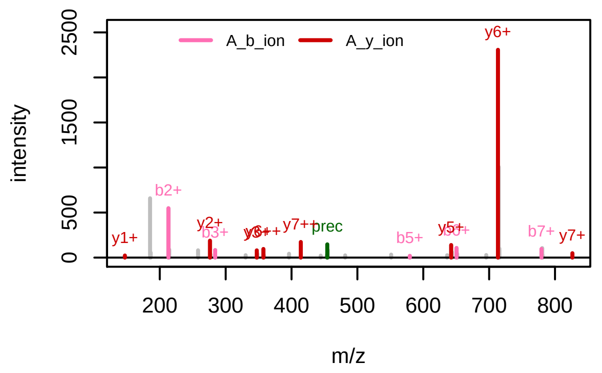

VLAKLAEK+XL_L

LVFTPKDGSSAK+XL_S

LVFTPKDGSSAK+XL_L
